# First detection of *Kudoa neothunni* in yellowfin tuna (*Thunnus albacares*) marketed in Portugal

**DOI:** 10.1017/S0031182026102236

**Published:** 2026-07

**Authors:** Caner Şirin, Paula Ramos, Camilo Ayra-Pardo, Ana Couto, Luís Filipe Rangel, Mário Quaresma, Maria João Santos

**Affiliations:** 1 CIIMAR/CIMAR LA, Interdisciplinary Centre of Marine and Environmental Research, University of Porto, Matosinhos, Portugal; 2 CIIMAR, Biology Department, Faculty of Sciences, University of Porto, Porto, Portugal; 3 Fatsa Faculty of Marine Sciences, Fisheries Technology Engineering, Ordu University, Ordu, Turkey; 4 IPMA, I.P., Portuguese Institute for the Ocean and Atmosphere, Algés, Portugal; 5 CIISA – Centre for Interdisciplinary Research in Animal Health, Faculty of Veterinary Medicine, University of Lisbon, Lisbon, Portugal

**Keywords:** *Kudoa neothunni*, molecular characterization, morphology, myxozoa, *Thunnus albacares*

## Abstract

This study reports the presence of *Kudoa neothunni* in yellowfin tuna (*Thunnus albacares*) landed in Portugal. The parasite was detected in fish harvested from the Indian and Pacific Oceans but not in the Atlantic Ocean specimens. Muscle samples collected from specimens were examined macroscopically and microscopically for parasitic infections. While no *Kudoa* cysts were observed macroscopically, microscopic analysis revealed *K. neothunni* in 10 fish (5 each from the Pacific and Indian Oceans), with a prevalence of 20.8% and 20%, respectively. Histological examinations confirmed the presence of *Kudoa* cysts in infected muscle tissue. Morphological analyses showed stellate spores with 6 polar capsules, and significant differences in spore length and polar capsule dimensions were found between isolates from the 2 oceanic regions. However, phylogenetic analysis based on small and large ribosomal subunit sequences indicated that both spores were consistent with *K. neothunni* and showed high (100%) similarity to previously published GenBank sequences. These results contribute to the current knowledge of *K. neothunni* occurrence in yellowfin tuna and indicate that infected specimens originating from the Indian and Pacific Oceans enter the European seafood market. Given that some *Kudoa* infections can induce *post-mortem* myoliquefaction and adversely affect fillet quality, this should be considered a potential concern.

## Graphical abstract

**Figure:**
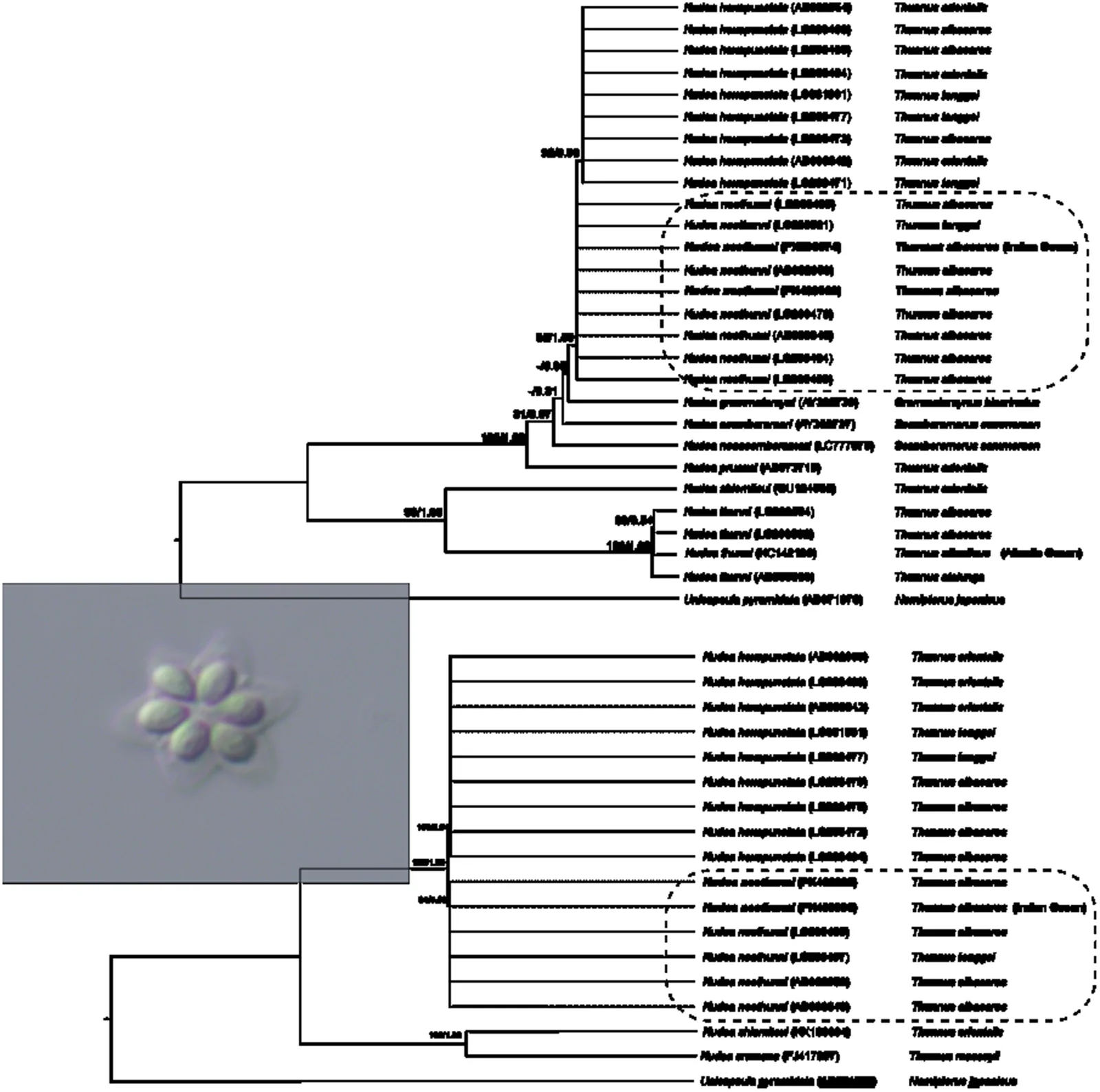

## Introduction

The myxozoan genus *Kudoa* Meglitsch, 1947, primarily comprises histozoic species and is among the most speciose parasite genera found in marine fish, with over a 100 species formally described (Eiras et al., 2014; Yurakhno, 2024; Whipps et al., 2025). Their spores are typically stellate, subquadrate or rounded-quadrangular and contain 2–13 polar capsules and shell valves (Lom and Dyková, 2006; Casal et al., 2019). Members of the genus infect a variety of host tissues, including nervous and visceral organs; however, the majority display myotropism, preferentially invading the host’s musculature (Eiras et al., 2014; Yurakhno, 2024). Several *Kudoa* species, including *Kudoa thyrsites* (Gilchrist, 1924), *Kudoa encrasicoli* Iglesias, Rangel, Fernández-Vázquez, Santos and García-Estévez, 2022; *Kudoa lateolabracis* (Yokoyama et al., 2004), *Kudoa musculoliquefaciens* (Mantsumoto, 1954); *Kudoa paniformis* Kabata and Whitaker, 1981; and *Kudoa pleurogrammi* (Kasai et al., 2016) are recognized as causative agent of *post-mortem* myoliquefaction in the muscle tissue of infected fish hosts (Mantsumoto, 1954; Yokoyama et al., 2004; Funk et al., 2008; Kasai et al., 2016; Bolin et al., 2021; Giulietti et al., 2024). Besides causing tissue damage, species such as *Kudoa iwatai* Egusa and Shiomitsu, 1983, *Kudoa thunni (*Matsukane et al., 2011) and *Kudoa trachuri (*Matsukane et al., 2011), can cause visible cysts to form in the muscle, which in turn can negatively affect the appearance of infected fish and reduce their market value (Matsukane et al., 2011; Bolin et al., 2021; Hoai et al., 2022). Beyond these detrimental effects on fish quality and commercial value, growing scientific evidence indicates that *Kudoa septempunctata* Matsukane; Sato; Tanaka; Kamata and Sugita-Konishi, 2010; and *Kudoa hexapunctata* (Yokoyama et al., 2014), are the species associated with gastrointestinal infections in humans, particularly in countries where raw fish is commonly consumed (Kawai et al., 2012; Yokoyama et al., 2014; Suzuki et al., 2015; Yahata et al., 2015; Takeuchi et al., 2016; Tachibana and Watari, 2021; Shamsi and Barton, 2024).

Yellowfin tuna, *Thunnus albacares* (Bonnaterre, 1788), is a migratory fish species occurring in tropical and temperate regions across the world’s 3 major oceans (Arrizabalaga et al., 2015). It is recognized as 1 of the 7 major commercial tuna species worldwide (FAO, 2020). According to FAO data, yellowfin tuna, with a catch of over 1.5 million tonnes, is the second most harvested tuna species worldwide after skipjack tuna, *Katsuwonus pelamis* (Linnaeus, 1758) (FAO, 2025). Yellowfin tuna also comprised 31% of the total catch of major commercial tuna species in 2023, reflecting its important role in global tuna fisheries (ISSF, 2025).

Current findings indicate that 4 species of *Kudoa* are known to infect yellowfin tuna. *Kudoa crumena* Iversen and Van Meter, 1967, was reported from *T. albacares* caught off the coast of North Carolina, USA (Moran et al., 1999). *K. thunni* was first reported from yellowfin tuna in Japan (Kasai et al., 2017) and was later recorded in Australia (Bolin et al., 2021) and Vietnam (Hoai et al., 2022). *K. hexapunctata* has been identified in *T. albacares* from the Pacific and Indian Ocean (Yokoyama et al., 2014; Kasai et al., 2017). The other *Kudoa* species identified in this fish species is *Kudoa neothunni* (Arai and Matsumoto, 1953), which was originally classified under the genus *Hexacapsula* (Arai and Matsumoto, 1953), but later reassigned to the *Kudoa* genus based on molecular analyses (Whipps et al., 2004). *K. neothunni* has also been reported from long-tail tuna, *Thunnus tonggol* (Bleeker, 1851), and Pacific bluefin tuna, *Thunnus orientalis* (Temminck and Schlegel, 1844), in Japanese waters (Abe and Maehara, 2013; Kasai et al., 2017). This species is particularly known in East Asian regions for causing *post-mortem* myoliquefaction (Arai and Matsumoto, 1953; Abe and Maehara, 2013; Li et al., 2013; Yokoyama et al., 2014).

In Portugal, *Kudoa* spp. have previously been recorded in *Thunnus obesus* (Lowe, 1839) from the Madeira Archipelago (Cavaleiro et al., 2021), and *K. hexapunctata* in tuna used in ready-made sashimi products (Ramos, 2020). However, data on the occurrence of *Kudoa* infections in tuna species of commercial importance to Portugal remain very limited. This study reports the presence of the *K. neothunni* in yellowfin tuna collected from different oceanic regions and landed in Lisbon, Portugal. Even though *K. neothunni* is not a newly identified species in the present host studied, its detection in a European country reflects its introduction into the local seafood market through imported fish originating from the Indian and Pacific Oceans. This finding is significant for the seafood industry due to the potential economic losses linked to *Kudoa* infections. In addition, given that some *Kudoa* species are zoonotic, the continuous monitoring of closely related *Kudoa* species in marketed yellowfin tuna may be important for public health.

## Materials and methods

### Fish sampling

**Figure 1.**
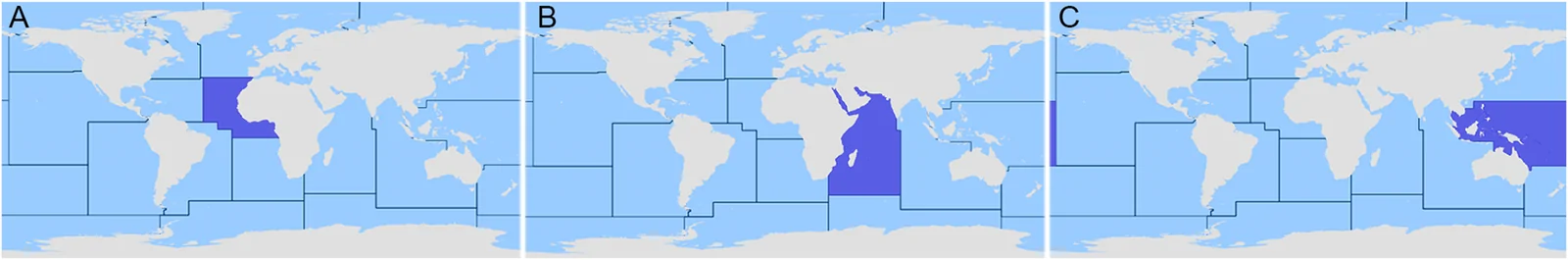
FAO fishing areas. (A) Area 34 Atlantic, Eastern Central, (B) Area 51, Indian Ocean, Western, (C) Area 71, Pacific, Western Central.

*Source*: European Commission, retrieved from https://fish-commercial-names.Ec.Europa.Eu/fish-names/fishing-areas_en.

In this study, muscle samples were collected from 72 yellowfin tuna (*T. albacares*) harvested between March and October 2020 from 3 FAO fishing areas (Figure 1) and landed in Lisbon, Portugal. The specimens originated from the Atlantic Ocean (FAO Area 34; *n* = 23; mean weight ± standard deviation (s.d.) = 30.6 ± 4.7 kg; harvested between September and October), the Indian Ocean (FAO Area 51; *n* = 25; mean weight ± s.d. = 49.9 ± 7.4 kg; harvested between April and June) and the Pacific Ocean (FAO Area 71; *n* = 24; mean weight ± s.d. = 39.3 ± 11.5 kg; harvested between March and April) (Domingues et al., 2021). Dorsal muscle portions excised from each fish were transported to the laboratory and stored at −20°C until the analysis. Prior to examination, frozen samples were thawed overnight at ≤5°C under refrigeration.

### Parasitological and morphological analyses

All muscle samples were initially examined macroscopically to detect the presence of *Kudoa* cysts. A small portion of muscle tissue (∼1 g) was taken from each tuna specimen, placed in a Petri dish, and finely minced in phosphate-buffered saline (PBS) using a sterile scalpel. The minced tissue was then compressed between 2 Petri dishes to obtain a homogenate. The expressed liquid was transferred to microcentrifuge tubes and centrifuged at 2100 *g* for 15 min. After removing the supernatant, the resulting pellet was resuspended in 100 µL of PBS. Wet mounts prepared from this suspension were examined under a light microscope at 400× and 1000× magnification to detect the presence of *Kudoa* spores. The observed spores were measured and photographed at 400× and 1000× magnification using a Zeiss Axiophot microscope equipped with a Flexicam C1 digital camera and LAS X software (Leica Microsystems). Figure labelling was carried out using Adobe Photoshop 2025 v26.11.2. Morphometric analyses were performed in accordance with Adlard et al. (2005). Measurements of spore width (SW), thickness (ST) and suture width (StW) were taken from spores in apical view, while spore length (SL), polar capsule length (PCL) and polar capsule width (PCW) were taken from spores in lateral view. For each parameter, 30 measurements were obtained. All measurements are given in micrometres and expressed as mean ± standard deviation (s.d.), with the range (minimum–maximum) in parentheses (*n* = 30 for each parameter). Additionally, in samples containing spores, 10 µL of the homogenate was transferred to a Neubauer chamber, and spores were counted under 400× magnification to estimate density (number of spores per gram of muscle).

Morphometric measurements were obtained from spores isolated from each region and subsequently compared to assess potential regional differences. The normality of the measurement data was assessed using the Shapiro–Wilk test. For normally distributed variables, differences between the 2 independent groups were analysed using Welch’s *t*-test. For non-normal data, the Mann–Whitney *U* test was applied. All statistical analyses were performed using R Studio (Posit team, 2025). Statistical significance was assessed using a threshold of *P* < 0.05, with exact *P*-values reported.

### Molecular analysis

Genomic DNA was extracted from the mixture obtained from infected muscle tissues using the NZY Tissue gDNA Isolation Kit (NZYtech, Lisbon, Portugal) according to the manufacturer’s protocol. The small subunit ribosomal DNA (SSU rDNA) and the large subunit ribosomal DNA (LSU rDNA) was amplified with myxozoan and *Kudoa*-specific primers (Table 1). Polymerase chain reactions (PCRs) were performed in 25 μL reaction volumes containing 0.2 μM of each primer, 1 × NZYTaq II Green Master Mix (NZYtech) and 2 µL of genomic DNA using an XT 96 Thermal Cycler (VWR). PCR amplifications were performed with initial denaturation at 95°C for 3 min, followed by 35 cycles of denaturation at 94°C for 50 s, annealing at 55°C for 50 s, extension at 72°C for 2 min and a final extension at 72°C for 7 min. Amplicons were electrophoresed on a 1% agarose gel in TAE buffer (NZYtech), stained with green safe premium (NZYtech), and positive PCR products were purified and sequenced using the same primers by STAB VIDA (Caparica, Portugal).

**Table 1.** Primers used for the amplification and sequencing of the small subunit (SSU) and large subunit (LSU) ribosomal DNA of *Kudoa neothunni* Table 1 long description.

Gene regions	Primers	Sequence (5′−3′)	References
18S rDNA	18e	CTGGTTGATCC TGCCAGT	Hillis and Dixon (1991)
	Kud6r	TCCAGTAGCTACTCA TCG	Whipps et al. (2003a)
	MyxospecF	TTCTGCCCTATCAAC TTGTTG	Fiala (2006)
	Myxgen4r	ACCTGTTATTGCCA CGCT	Kent et al. (2000)
	Kud2f	TGAATGTTATAGCAT GGAA	Whipps et al. (2003a)
	Kud6f	TCACTATCGGAATG AACG	Whipps et al. (2003a)
	18R	CTACGGAAACCTTGT TACG	Whipps et al. (2003b)
28S rDNA	ZX-1	ACCCGCTGAATTTAA GCATAT	Waeschenbach et al. (2007)
	1500R	GCTATCCTGAGGGAA ACTTCG	Waeschenbach et al. (2007)
	900F	CCGTCTTGAAACAC GGACCAAG	Waeschenbach et al. (2007)
	Kud2400fR	GATGCTTGCCACTCG TAAAG	Shin et al. (2016)

### Phylogenetic analysis

The partial sequences obtained from PCR amplifications were assembled with BioEdit software (Hall, 1999). Sequence similarity searches were subsequently performed through BLASTn against the GenBank nucleotide database (https://blast.ncbi.nlm.nih.gov/Blast.cgi). A total of 27 SSU rDNA and 17 LSU rDNA sequences with more than 99% nucleotide identity to the isolates obtained in this study, together with sequences of *Kudoa* species previously reported from tuna hosts, were included in the phylogenetic analysis. The species *Unicapsula pyramidata* (Naidenova and Zaika, 1970) (AB971675) was used as the out-group in both phylogenetic analyses. The sequences were aligned on MAFFT online server (https://mafft.cbrc.jp/alignment/server/) with the FFT-NS-i strategy (Katoh et al., 2002), and ambiguous nucleotide positions and gaps were removed using Gblocks 0.91b on the Phylogeny.fr server (http://phylogeny.lirmm.fr/phylo_cgi/one_task.cgi?task_type=gblocks) (Castresana, 2000; Dereeper et al., 2008).

Phylogenetic trees were constructed using maximum likelihood (ML) and Bayesian inference (BI) methods. ML analysis was performed in MEGA12 (Kumar et al., 2024) using the general time-reversible (GTR) with gamma-distributed rate (+G), which was selected based on the lowest corrected Akaike Information Criterion value, with 1000 bootstrap replicates. BI analysis was conducted in MrBayes v.3.2.7 (Ronquist et al., 2012) using the GTR + G nucleotide substitution model. The Markov chain Monte Carlo analysis was run for 1 million generations with 2 independent runs and 4 chains per run. Trees were sampled every 100 generations and the first 25% of samples were discarded as burn-in. The consensus trees were visualized using FigTree v1.4.4 (https://tree.bio.ed.ac.uk/software/figtree/) and edited in Adobe Illustrator 2025 v29.8.3. The genetic distance was calculated in MEGA12 using the *P*-distance and number of differences method (Kumar et al., 2024).

### Histological analysis

Muscle tissues from infected tuna fish were cut into small pieces and fixed in 10% neutral buffered formalin at room temperature for 24 h and then transferred to 70% ethanol. The fixed samples were processed using a tissue processor (Thermo Fisher Scientific, USA) and embedded in paraffin. Paraffin blocks were sectioned at 5 μm thickness and mounted on glass slides. The sections were stained with haematoxylin and eosin (H&E), and slides were mounted using the Entellan mounting medium. Prepared slides were examined under a light microscope and photographed using a Zeiss Axiophot microscope equipped with a Flexicam C1 digital camera and LAS X software (Leica Microsystems).

## Results

Morphological features and molecular data of the myxospores supported the identification of the detected species as *K. neothunni*. No macroscopic signs of *Kudoa* cysts or muscle softening were observed during macroscopic examinations of muscle samples. However, microscopic analysis revealed *Kudoa* spores in 10 out of 72 fish, comprising 5 specimens each from the Pacific Ocean (5/24, 20.8% prevalence) and Indian Ocean (5/25, 20% prevalence). No infections were detected in samples originated from the Atlantic Ocean (0/23, 0% prevalence).

### Morphological description of *K. neothunni*

**Figure 2.**
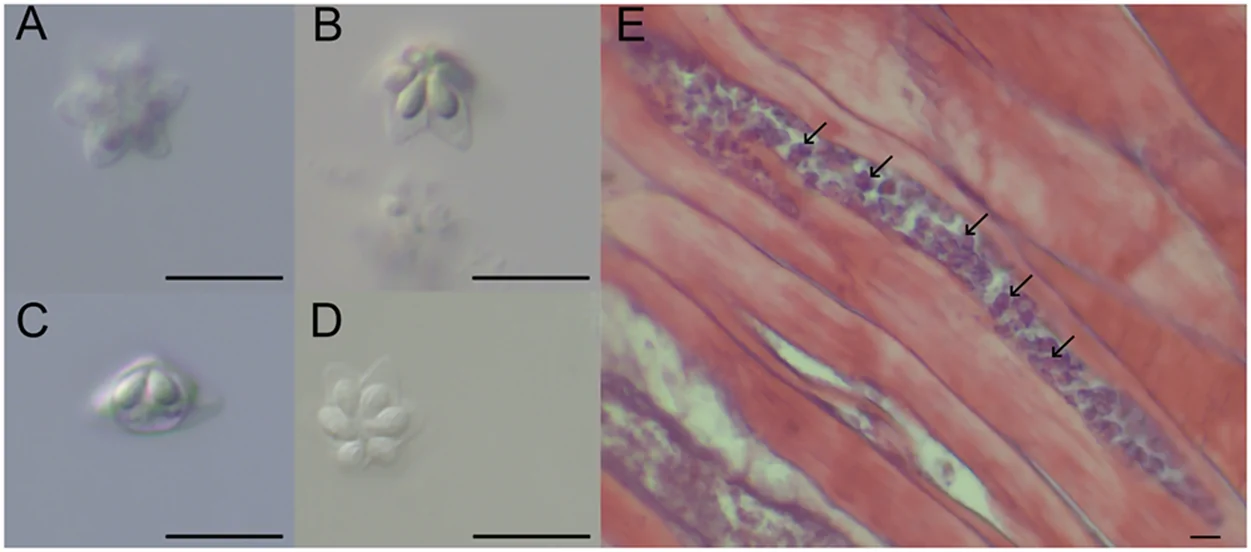
Wet mount preparation of fresh *Kudoa neothunni* infected muscle tissue of the yellowfin tuna were examined under a light microscope (A–D), Apical view of *K. Neothunni* spores from the Pacific (A–B) and the Indian isolate (D), lateral view of *K. Neothunni* spores from the Indian isolate (C), H&E-stained histological sections of infected muscle tissue from the Indian sample, with *K. Neothunni* spores indicated by arrows (E) (Scale bar 10 µm).

Myxospores of *K. neothunni* from both isolates were stellate in apical view with clearly visible suture lines and 6 equal, pointed spore valves, each containing a single polar capsule. Myxospores isolated from Pacific Ocean specimens measured 6.5 ± 0.3 (6.0–7.1) µm in length, 11.7 ± 0.4 (11.1–12.6) µm in width, 10.3 ± 0.4 (9.3–11.1) µm in thickness and 6.7 ± 0.4 (5.7–7.4) µm in StW. The polar capsules of the Pacific isolate were pyriform, measuring 3.8 ± 0.2 (3.5–4.3) µm in length and 2.3 ± 0.2 (2.1–3.0) µm in width. Myxospores isolated from Indian Ocean specimens measured 6.7 ± 0.3 (6.1–7.3) µm in length, 11.7 ± 0.4 (10.8–12.4) µm in width, 10.1 ± 0.5 (8.9–10.8) µm in thickness and 6.7 ± 0.4 (5.9–7.6) µm in StW. Their polar capsules were pyriform and measured 3.7 ± 0.2 (3.4–4.1) µm in length and 2.2 ± 0.1 (2.1–2.7) µm in width (Figure 2; Table 2). The number of polar filament coils could not be determined in any wet mount preparations.

**Table 2.** Morphological comparison of *Kudoa neothunni* and related species in tuna fishes Table 2 long description.

Parasite	Host	Locality	*n*	SW	ST	StW	SL	PCL	PCW	References
*K. neothunni*	*Thunnus albacares*	Pacific Ocean	30	11.7 ± 0.4	10.3 ± 0.4	6.7 ± 0.4	6.5 ± 0.3	3.8 ± 0.2	2.3 ± 0.2	This study
				(11.1−12.6)	(9.3−11.1)	(5.7 – 7.4)	(6.0 – 7.1)	(3.5 – 4.3)	(2.1 – 3.0)	
		Indian Ocean	30	11.7 ± 0.4	10.1 ± 0.5	6.7 ± 0.4	6.7 ± 0.3	3.7 ± 0.2	2.2 ± 0.1	
				(10.8−12.4)	(8.9−10.8)	(5.9 – 7.6)	(6.1 – 7.3)	(3.4 – 4.1)	(2.1 – 2.7)	
*K. neothunni*	*Thunnus albacares*	Pacific Ocean, Philippines	15	10.6	9.7	8.1	7.6	3.8	2.0	Li et al. (2013)
				(9.5−11.4)	(8.9−10.9)	(7.3−8.6)	(7.3−7.7)	(3.6−4.1)	(1.8−2.3)	
*K. neothunni*	*Thunnus albacares*	Japan	30	10.2	6.8	–	–	–	–	Abe and Maehara (2013)
				(9.0−12.0)	(6.0−7.5)					
				10.3						
				(9.0−12.0)						
*K. neothunni*	*Thunnus albacares*	Bunda Sea	–	11.0	7.1	–	6.2	2.5	1.6	Arai and Matsumoto (1953)
				(9.1−13.0)	(5.9−8.7)		(5.3−7.3)	(2.0−3.1)	(1.3−2.1)	
*K. neothunni*	*Thunnus albacares*	Vanuatu	20	11.7 ± 0.7	9.7 ± 0.5	7.4 ± 0.3	7.0 ± 0.4	3.5 ± 0.7	2.0 ± 0.1	Yokoyama et al. (2014)
				(10.7−12.9)	(8.6−10.5)	(6.8−7.9)	(6.3−7.5)	(2.4−4.5)	(1.8−2.2)	
		South Korea	20	11.0 ± 0.5	9.2 ± 0.5	7.3 ± 0.4	6.6 ± 0.4	3.5 ± 0.4	1.9 ± 0.1	
				(9.7−11.5)	(8.4−10.0)	(6.4−7.8)	(5.9−7.4)	(2.4−4.5)	(1.7−2.2)	
		Philippines	20	11.8 ± 0.7	10.4 ± 0.4	7.9 ± 0.6	–	–	1.7 ± 0.1	
				(11.1−12.7)	(9.7−10.8)	(6.9−8.5)			(1.6−1.9)	
		Fiji	20	12.0 ± 0.5	10.1 ± 0.5	7.4 ± 0.4	6.5 ± 0.4	3.5 ± 0.4	2.1 ± 0.1	
				(11.0−12.7)	(9.3−11.0)	(6.9−8.5)	(5.9−7.4)	(2.8−4.1)	(1.8−2.3)	
		–	20	11.5 ± 0.6	9.7 ± 0.6	7.6 ± 0.2	6.8 ± 0.3	3.9 ± 0.4	2.0 ± 0.2	
				(10.6−12.1)	(9.1−10.5)	(7.4−7.9)	(6.3−7.7)	(2.9−4.3)	(1.7−2.1)	
*K. neothunni*	*Thunnus tonggol*	East China Sea off Nagasaki, Japan	20	12.1	10.7	–	8.1	4.8	2.0	Kasai et al. (2017)
				(11.0−13.0)	(9.2−12.0)		(7.5−8.9)	(3.9−5.7)	(1.6−2.4)	
*K. neothunni*	*Thunnus orientalis*	Nagasaki, Japan	30	10.2	6.8	–	–	–	–	Abe and Maehara (2013)
				(9.0−12.0)	(6.0−7.5)					
				10.3						
				(9.0−12.0)						
*K. thunni*	*Thunnus albacares*	Coral Sea	60	9.4	8.4	7.6	6.4	2.6	1.7	Bolin et al. (2021)
				(7.8−10.4)	(7.1−9.5)	(6.1−9.3)	(5.4−7.2)	(2−3)	(1.2−2.4)	
*K. thunni*	*Thunnus albacares*	Vietnam	10	9.4	8.1	–	6.3	(2.5)	2.0	Hoai et al. (2022)
				(9.0−9.8)	(7.2−8.5)		(5.8−6.9)	(2.2−2.7)	(1.8−2.2)	
*K. hexapunctata*	*Thunnus albacares*	Pacific Ocean	20	10.2 ± 0.4	9.0 ± 0.3	8.0 ± 0.3	7.1 ± 0.5	3.2 ± 0.6	1.9 ± 0.2	Yokoyama et al. (2014)
				(9.3−11.0)	(8.4−9.4)	(7.3−8.4)	(6.4−8.1)	(2.3−4.1)	(1.6−2.2)	
		Indian Ocean	20	9.7 ± 0.3	8.5 ± 0.3	7.3 ± 0.3	7.0 ± 0.4	3.6 ± 0.5	1.9 ± 0.2	
				(9.3−10.4)	(7.9−9.0)	(6.7−7.7)	(6.0−7.5)	(2.3−4.1)	(1.5−2.1)	
		Indian Ocean	20	9.8 ± 0.4	8.7 ± 0.6	7.8 ± 0.6	7.2 ± 0.3	3.6 ± 0.4	1.9 ± 0.2	
				(9.1−10.7)	(7.8−10.3)	(7.0−9.3)	(6.7−7.4)	(2.8−4.1)	(1.5−2.3)	

SW spore width, ST suture thickness, StW suture width, SL spore length, PCL polar capsule length, PCW polar capsule width. Measurements are given in micrometres as mean ± standard deviation (s.d.), and minimum–maximum ranges are shown in parentheses. *N* = number of spores measured

Morphologically, no statistically significant differences were found between the parasite specimens of the Pacific Ocean and the Indian Ocean in SW width (*P* = 0.672), ST (*P* = 0.116) and StW (*P* = 0.402). However, statistically significant differences were detected in SL (*P* = 0.014) and PCL (*P* = 0.029). Additionally, a significant difference in PCW between the 2 regions was observed (*P* = 0.019). Microscopic examination showed that the average spore density was 1.4 × 10^4^ spores per gram of muscle in Pacific isolates, whereas 3.8 × 10^3^ spores per gram were detected in Indian isolates. Histological examinations revealed that *K. neothunni* cysts were localized within the muscle tissue of yellowfin tuna, situated inside the muscle fibres as long, non-wide cysts. In the infected muscle tissues, parasite spores were stained purple in H&E staining (Figure 2E).

### Molecular analyses

A total of 2 partial SSU rDNA sequences (1694 bp) and 2 partial LSU rDNA sequences (1487 bp) were generated from the samples obtained from the Pacific and Indian Oceans. All SSU and LSU rDNA sequences were 100% identical between each isolate and have been deposited in GenBank under the accession numbers PX488332, PX596674 for SSU rDNA and PX488395, PX488396 for LSU rDNA, for the Pacific and Indian Ocean spores, respectively.

BLASTn searches of the SSU rDNA sequences showed 100% nucleotide identity with *K. neothunni* isolates from *Thunnus tonggol* (Bleeker, 1851) in Japan (LC200497-LC200501) (Kasai et al., 2017) as well as with isolates from *T. albacares* collected in Japan (AB693049, AB698884) (Abe and Maehara, 2013; Li et al., 2013), in Indonesia (LC200488) (Kasai et al., 2017) and in Vanuatu (AB902958) (Yokoyama et al., 2014). The sequences also showed 99.94% nucleotide identity to isolates from *T. albacares* in the Philippines (LC200484, LC200479) and Thailand (LC200495), and 99.88% identity to an isolate from the Philippines (LC200485) (Kasai et al., 2017).

**Figure 3.**
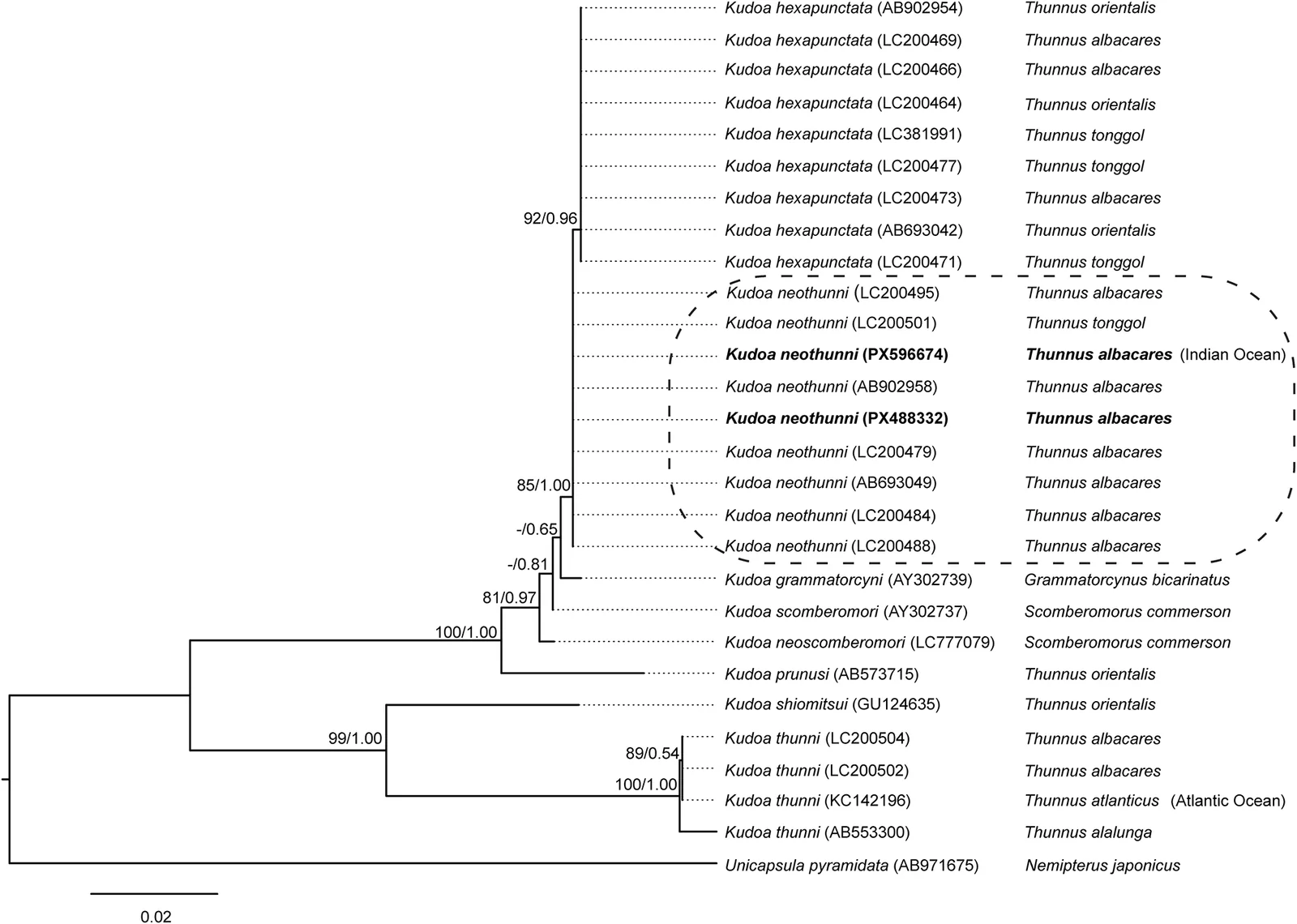
Maximum likelihood phylogenetic tree for small subunit ribosomal DNA sequences of *Kudoa neothunni* and other selected sequences. The numbers at the nodes represent Maximum likelihood bootstrap values/Bayesian inference posterior probabilities. GenBank accession numbers of species are given in parentheses. Bold text indicates isolates identified in this study. Bootstrap values less than 70 are represented by dashes. The scale bar represents the number of nucleotide substitutions per site. All parasite species originate from hosts in the Pacific Ocean, unless otherwise indicated. Species from other regions are specified in parentheses. Figure 3 long description.

Additionally, BLASTn searches revealed that the SSU rDNA sequences obtained in this study shared high nucleotide identity (99.76–99.88%) with several *K. hexapunctata* isolates from *T. tonggol* (LC200477, LC200471, LC381991), *Thunnus orientalis* (Temminck and Schlegel, 1844) (LC200462-LC200465, AB902954, AB693042) (Li et al., 2013; Yokoyama et al., 2014; Kasai et al., 2017) and *T. albacares* (LC200466, LC200469, LC200473) (Kasai et al., 2017). Lower levels of SSU rDNA identity were also observed with several other *Kudoa* species, including 99.52% identity with *Kudoa grammatorcyni* (Adlard et al., 2005) (AY302739), 99.46% with *Kudoa scomberomori* (Adlard et al., 2005) (AY302737) (Whipps et al., 2004), 99.29% with *Kudoa neoscomberomori* (Li et al., 2024) (LC777079) (Li et al., 2024) and 99.04% with *Kudoa konishiae* (Sakai et al., 2018) (LC316965) (Sakai et al., 2018) (Figure 3). Analysis of the SSU rDNA sequence (1,643 bp) revealed only 2 nucleotide differences and a genetic distance of 0.001 between the isolates obtained in this study and *K. hexapunctata*, the most similar species.

**Figure 4.**
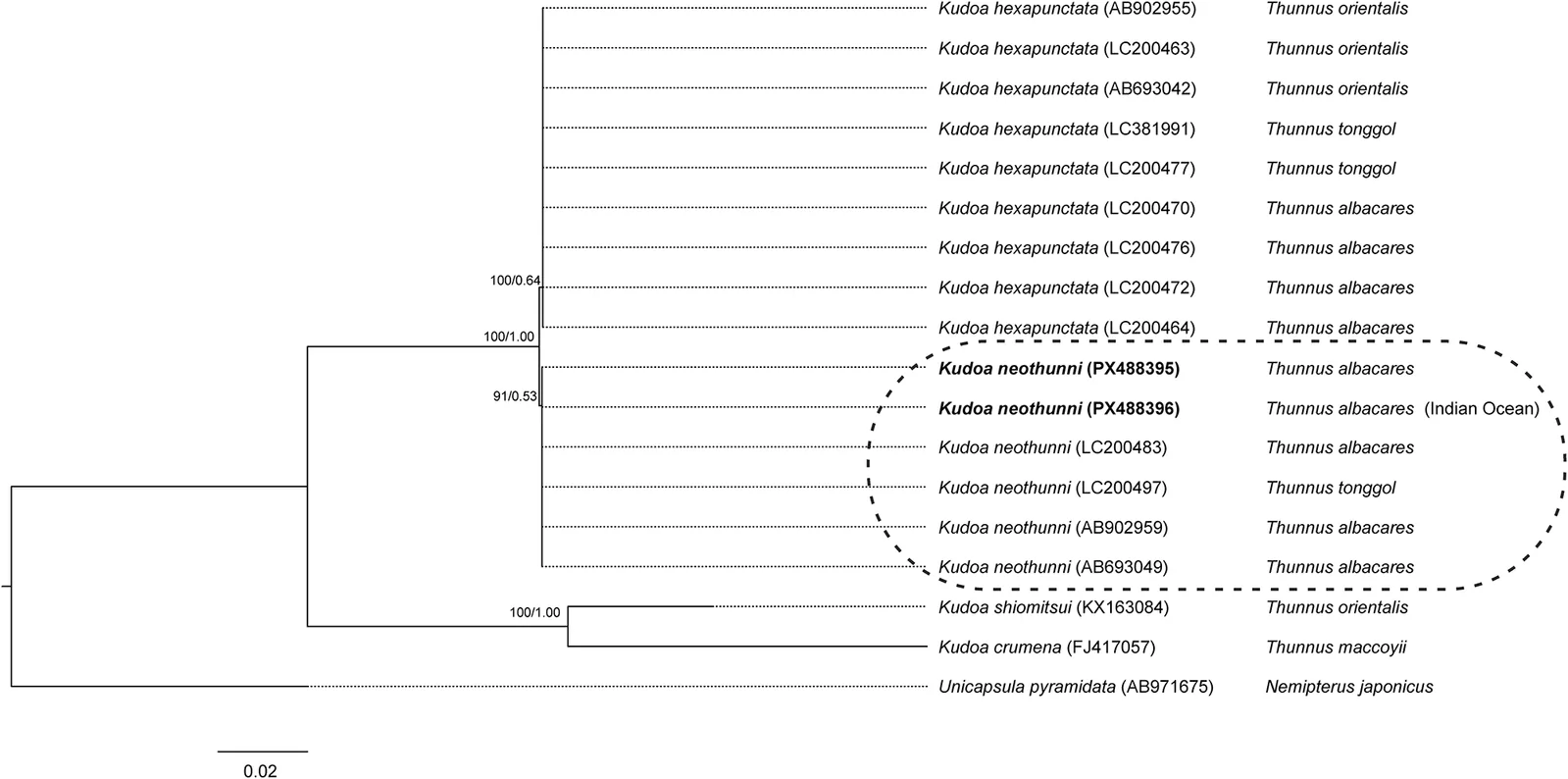
Maximum likelihood phylogenetic tree for large subunit ribosomal DNA sequences of *Kudoa neothunni* and other selected sequences. The numbers at the nodes represent Maximum likelihood bootstrap values/Bayesian inference posterior probabilities. GenBank accession numbers of species are given in parentheses. Bold text indicates isolates identified in this study. The scale bar represents the number of nucleotide substitutions per site. All parasite species originate from hosts in the Pacific Ocean, unless otherwise indicated. Species from other regions are specified in parentheses. Figure 4 long description.

Phylogenetic analysis based on the SSU rDNA sequences (Figure 3) showed that the 2 *K. neothunni* isolates obtained in the present study clustered together with other *K. neothunni* sequences retrieved from GenBank with moderate nodal support (85/1.00, ML bootstrap/Bayesian posterior probability). However, the *K. neothunni* sequences did not form a monophyletic clade on their own. Instead, they grouped outside the well-supported *K. hexapunctata* clade (92/0.96), which comprised sequences derived from different tuna hosts. The LSU rDNA sequences obtained in this study showed high nucleotide identity with several *K. neothunni* isolates available in GenBank. The highest identity values (99.93%) were observed with isolates derived from *T. albacares* in Vanuatu (AB902959) (Yokoyama et al., 2014), Japan (AB693049) (Li et al., 2013), the Philippines (LC200483) and an isolate from *T. tonggol* in Japan (LC200500) (Kasai et al., 2017). Additionally, slightly lower identity values (99.87%) were also obtained for 4 isolates from *T. tonggol* in Japan (LC200497–LC200499, LC200501) (Kasai et al., 2017). In addition to identity with *K. neothunni* isolates, BLASTn search of the LSU rDNA sequences showed lower identity values (99.12–99.20%) with several *K. hexapunctata* isolates available in GenBank (Figure 4). Based on the 28S rDNA sequence, 7 nucleotide differences and a genetic distance of 0.005 across 1491 base pairs were detected between the present isolates and *K. hexapunctata* isolates.

Phylogenetic analysis of the LSU rDNA sequences (Figure 4) showed that the 2 *K. neothunni* formed a lineage with moderate nodal support (91/0.53), together with other *K. neothunni* sequences. This *K. neothunni* lineage was separated from the *K. hexapunctata* clade (100/0.64). Together, these 2 lineages formed a strongly supported clade (100/1.00). *Kudoa shiomitsui* Egusa and Shiomitsu, 1983, and *K. crumena* clustered as more distantly related species.

## Discussion

This study presents the morphological and molecular identification of *K. neothunni* in yellowfin tuna, *T. albacares*, and reports the first recorded occurrence of this myxosporean species in the Indian Ocean and in fish from Pacific and Indian Oceans landed in an European country, in Portugal.

The morphometric measurements of *K. neothunni* obtained in this study are generally consistent with previously reported morphometric values for the species. However, minor differences were observed both between the 2 present isolates and in comparison, with those described in earlier studies (Table 2). These morphological differences are relatively limited and may indicate local adaptation among geographically distinct *K. neothunni* populations or may result from changes associated with spores obtained from frozen/thawed tuna rather than from fresh specimens. Even though the statistical analyses showed significant differences in some morphometric parameters between specimens from different oceans, these differences do not appear to have truly discriminatory value. Because measurement ranges overlap considerably across samples, spore morphometry alone is not sufficient to assign an individual specimen to a specific geographic origin. The current *K. neothunni* spores isolated from *T. albacares* exhibited some morphological differences when compared to isolates obtained from other tuna species. *K. neothunni* spores isolated from long-tail tuna, *T. tonggol*, were found to have longer spores and polar capsules, but smaller PCW than the present specimens (Kasai et al., 2017). In contrast, the spores obtained in this study had greater SW and ST compared to those reported from *T. orientalis* by Abe and Maehara (2013). These variations may be influenced by host-specific factors as well as geographical differences in environmental conditions. Additionally, the spore measurements from the host *T. orientalis* (initially misidentified as *T. thynnus* (Kasai et al., 2017)) reported by Li et al. (2013) are also smaller than current isolates. However, it is important to note that the *K. neothunni* isolate (AB693042) described by Li et al. (2013) was subsequently synonymized with *K. hexapunctata* (Yokoyama et al., 2014). Therefore, the comparison of *K. neothunni* spores in this study with those reported by Li et al. (2013) is effectively a comparison between 2 distinct species, which explains the observed morphological differences. Given the methodological limitations of morphometric interpretations and factors such as spore orientation and inter-observer variability, the small differences observed are unlikely to reflect true biological variation. Therefore, morphological findings should be considered supportive of molecular data.

The morphometric characteristics of the *K. neothunni* isolates obtained in this study were notably different from those of *K. hexapunctata*, a zoonotic species described by Yokoyama et al. (2014) in yellowfin tuna. In particular, the SW and ST of *K. neothunni* were larger than those of *K. hexapunctata*. In contrast, *K. hexapunctata* exhibits longer spores and a wider suture line, indicating a more elongated spore morphology. In another study on *K. hexapunctata* from yellowfin tuna (Arai et al., 2020) and *K. hexapunctata* isolated tuna muscles from ready-made sashimi products in centre Portugal (Ramos, 2020), morphological or molecular data were not provided, and thus no comparison could be made.

Extensive taxonomic analyses have shown that LSU rDNA is more informative than SSU rDNA, both within the genus *Kudoa* and in the broader Myxosporea phylogeny (Whipps and Kent, 2006; Bartošová et al., 2009). This pattern has also been critical in resolving morphologically similar, closely related species. Yokoyama et al. (2014) reported that there were only a few nucleotide differences in the 18S rDNA between *K. neothunni* and *K. hexapunctata*, whereas significantly greater differentiation was observed in the 28S rDNA; this difference supported the recognition of *K. hexapunctata* as a separate species. Kasai et al. (2017) further confirmed this finding in a larger dataset including isolates from 3 *Thunnus* species. In the present study, SSU rDNA showed a difference of only 2 nucleotides (genetic distance 0.001), while LSU rDNA showed a difference of 7 nucleotides (genetic distance 0.005). This indicates that the LSU region offers approximately fivefold greater resolving power for distinguishing these closely related taxa. However, since the obtained LSU rDNA sequence was limited to 1487 bp, sequencing longer LSU regions in further studies will allow for a more complete characterization of interspecific variation.

The SSU rDNA and LSU rDNA sequences of the *K. neothunni* isolates obtained in this study clustered within a well-supported monophyletic clade together with previously reported *K. neothunni* sequences isolated from yellowfin tuna in Indonesia, Papua New Guinea, Thailand and the Philippines (Kasai et al., 2017); Japan (Abe and Maehara, 2013; Li et al., 2013); and Vanuatu (Yokoyama et al., 2014), as well as from long-tail tuna fished in the East China Sea off Nagasaki, Japan (Kasai et al., 2017). This suggests a broad host range highlights the need for monitoring in other tuna and tuna-like species. Moreover, although the parasite had previously been reported only from the Asia–Pacific region, its detection in the Indian Ocean fish specimens entering the European market suggests an expansion of its known geographical distribution beyond these regions. This evidences the need for careful analysis of the fish stressed by EFSA (EFSA BIOHAZ Panel et al., 2024).

During the examinations, infections were detected only in specimens originating from the Pacific and Indian Oceans, while no infections were observed in samples from the Atlantic Ocean. Although previous studies have reported this parasite in tuna captured during different seasons and across various years (Arai and Matsumoto, 1953; Abe and Maehara, 2013; Li et al., 2013; Yokoyama et al., 2014; Kasai et al., 2017), infected specimens in this study were detected only during the early spring and summer months, owing to the limited sampling period. The fact that *Kudoa* infection was not detected in fish from the Atlantic Ocean may be attributed to several factors. One possibility is that the parasite is indeed absent from this system; however, the sample size of this study (*n* = 23 fishes) is too small to support this with confidence. The other is that the parasite may occur but is not detected during examination due to factors such as low prevalence, seasonality or differences in susceptibility to infection among hosts. In either case, broader sampling is needed to reach a reliable conclusion.

*K. neothunni* was detected in 2 tuna samples of unidentified species that were collected from foodborne disease cases (Arai et al., 2020). However, the direct association of *K. neothunni* with adverse effects on human health has not yet been demonstrated, whereas its close phylogenetic relative *K. hexapunctata* has been reported to cause gastrointestinal symptoms in humans in Japan (Suzuki et al., 2015; Tachibana and Watari, 2021; Takashima et al., 2021). Previous studies have also associated *K. neothunni* with post-harvest myoliquefaction (Arai and Matsumoto, 1953; Li et al., 2013). However, in the present study, no macroscopic signs of cyst formation or muscle tissue degradation were observed in any of the specimens. This discrepancy may be attributed to low infection intensity and infections stage. Although no *post-mortem* muscle degradation or softening was observed, the presence of *Kudoa* spores may still pose a latent risk to product quality.

## Conclusion

This study highlights the importance of identifying the fishing grounds for yellowfin tuna sold in Portugal, a southwest country of the European Union. Although zoonotic potential of *K. neothunni* has not been confirmed, its presence in commercially marketed tuna highlights the need for continued monitoring of product quality and food safety, also stressed by EFSA. The occurrence of cysts in the muscle can reduce product quality, potentially resulting in *post-mortem* myoliquefaction of the fish, which will surely cause economic losses. However, the limited sample size and short sampling period make it difficult to reach a definitive conclusion regarding the absence of *K. neothunni* in the Atlantic Ocean. Further sampling across different seasons and geographic regions would help clarify whether this parasite is absent or rare in yellowfin tuna populations distributed across the Atlantic Ocean.

## Data Availability

The sequence data generated in this study have been deposited in the publicly accessible GenBank database under accession numbers PX488332, PX596674, PX488395, and PX488396. Two histological slides were deposited in CIIMAR Parasitology Collection with the access numbers CIIMAR.2026.92-CIIMAR.2026.93.

## Figure 3 Long description

The diagram is a maximum likelihood phylogenetic tree for small subunit ribosomal DNA sequences. It is oriented vertically, with branches extending from the bottom to the top. The tree includes several species of Kudoa, with GenBank accession numbers in parentheses. Bold text indicates isolates identified in this study. The numbers at the nodes represent maximum likelihood bootstrap values and Bayesian inference posterior probabilities. Bootstrap values less than 70 are shown as dashes. The scale bar at the bottom left represents the number of nucleotide substitutions per site, marked as 0.02. The tree shows Kudoa species originating from hosts in the Pacific Ocean, with some species from other regions specified in parentheses. The tree highlights the evolutionary relationships among the species, with Kudoa neothunni and Kudoa hexapunctata being closely related to Thunnus species, such as Thunnus albacares and Thunnus tonggol. The diagram also includes other species like Scomberomorus commerson and Nemipterus japonicus, showing their evolutionary connections.

Navigate back to Figure 3.

## Figure 4 Long description

The diagram is a phylogenetic tree illustrating the relationships among large subunit ribosomal DNA sequences. The tree is oriented vertically, with branches extending from left to right. At the base, the tree splits into two main branches. The left branch leads to a single sequence labelled ‘Unicapsula pyramidata (AB971675)’ associated with ‘Nemipterus japonicus.’ The right branch further divides into multiple sequences, primarily labelled as ‘Kudoa hexapunctata’ with various GenBank accession numbers, all associated with different ‘Thunnus’ species. Notably, two sequences labelled in bold as ‘Kudoa neothunni (PX488395)’ and ‘Kudoa neothunni (PX488396)’ are highlighted within a dashed oval, indicating their association with ‘Thunnus albacares’, with PX488396 from the Indian Ocean. The scale bar at the bottom represents the number of nucleotide substitutions per site, marked as ‘0.02.’ Bootstrap values are indicated at the nodes, with values such as ‘100/1.00’ and ‘91/0.53’ shown, representing maximum likelihood bootstrap values and Bayesian inference posterior probabilities, respectively.

Navigate back to Figure 4.

## Table 1 Long description

The table provides primer sets used to amplify and sequence two ribosomal DNA gene regions in Kudoa neothunni: 18S rDNA and 28S rDNA. For each primer, it lists the primer name, the nucleotide sequence written from five prime to three prime, and the literature reference. The 18S rDNA section includes seven primers: 18e, Kud6r, MyxospecF, Myxgen4r, Kud2f, Kud6f, and 18 R, with references spanning Hillis and Dixon, Whipps and colleagues, Fiala, and Kent and colleagues. The 28S rDNA section includes four primers: ZX minus 1, 1500 R, 900 F, and Kud2400fR, cited to Waeschenbach and colleagues and Shin and colleagues. Primer sequences vary in length across entries, reflecting different target sites within each gene region. The table is a reference list and does not report amplification performance, specificity, or experimental conditions, so suitability depends on the user’s assay design and sample context.

Navigate back to Table 1.

## Table 2 Long description

The table compiles spore measurements for several Kudoa species found in tuna hosts, listing sample size, locality, and spore dimensions (width, thickness, suture width, length, and polar capsule size) in micrometres with ranges. For Kudoa neothunni in yellowfin tuna, spore width is consistently around the low to mid 11 micrometre range in the Pacific and Indian oceans, with spore thickness near about 10 micrometres and spore length commonly around the mid 6 micrometre range; polar capsules are roughly around 3.5 to 3.9 micrometres long and about 2 micrometres wide. Published records for Kudoa neothunni vary by study and locality, including a Philippines record with smaller width but larger suture width and longer spore length, and older or partial records from Japan and the Bunda Sea with several missing fields. Kudoa neothunni is also reported from other tuna species, including Thunnus tonggol off Japan, where spore width and thickness are among the largest listed and spore length is notably longer than most yellowfin records. Kudoa thunni in yellowfin tuna (Coral Sea and Vietnam) shows smaller spore width and smaller polar capsules than most Kudoa neothunni entries. Kudoa hexapunctata in yellowfin tuna has spore widths near about 10 micrometres and polar capsule widths near about 2 micrometres, overlapping with Kudoa neothunni but generally below it in width and thickness. Comparisons should be interpreted cautiously because sample sizes differ by row, some measurements are missing, and methods may vary across references.

Navigate back to Table 2.
